# Preparation and Characterization of Polysulfone Membranes Reinforced with Cellulose Nanofibers

**DOI:** 10.3390/polym14163317

**Published:** 2022-08-15

**Authors:** Reema H. Alasfar, Viktor Kochkodan, Said Ahzi, Nicolas Barth, Muammer Koç

**Affiliations:** 1 Division of Sustainable Development, College of Science and Engineering, Hamad Bin Khalifa University, Qatar Foundation, Education City, Doha P.O. Box 34110, Qatar; 2 Qatar Environment and Energy Research Institute, Hamad bin Khalifa University, Qatar Foundation, Education City, Doha P.O. Box 34110, Qatar; 3 ICUBE Laboratory—CNRS, University of Strasbourg, 67000 Strasbourg, France

**Keywords:** polymeric membranes, polysulfone, cellulose nanofibers, mechanical properties, pore morphology

## Abstract

The mechanical properties of polymeric membranes are very important in water treatment applications. In this study, polysulfone (PSF) membranes with different loadings of cellulose nanofibers (CNFs) were prepared via the phase inversion method. CNF was characterized through transmission electron microscopy (TEM) and scanning electron microscopy (SEM). The pore morphology, mechanical properties, membrane performance and hydrophilicity of pure PSF membranes and PSF/CNF membranes were investigated. The changes in membrane pore structure with the addition of different CNF contents were observed using SEM images. It was shown that the calculated membrane pore sizes correlate with the membrane water fluxes. The pure water flux (PWF) of fabricated membranes increased with the addition of CNFs into the PSF matrix. It was shown that the optimal CNF loading of 0.3 wt.% CNF improved both the elastic modulus and yield stress of the PSF/CNF membranes by 34% and 32%, respectively (corresponds to values of 234.5 MPa and 5.03 MPa, respectively). This result indicates a strong interfacial interaction between the PSF matrix and the reinforced nanofibers. The calculated compaction factor (CF) showed that the membrane resistance to compaction could be improved with CNF reinforcement. Compared to pure PSF membrane, the hydrophilicity was significantly enhanced with the incorporation of 0.1 wt.%, 0.2 wt.% and 0.3 wt.% CNF, as shown by the water contact angle (WCA) results. It can be concluded that CNFs are homogeneously dispersed within the PSF matrix at CNF loading less than 0.5 wt.%.

## 1. Introduction

Currently, polymeric membranes are the most commonly used membranes in water treatment applications due to their wide commercial availability, low cost and high efficiency [1]. Polysulfone (PSF) is one of the widely used polymers for the fabrication of pressure-driven membranes due to its good mechanical properties, chemical resistance and thermal stability [2,3]. For the membrane applications in water treatment, the importance of having good mechanical strength is due to the fact that, at operating pressure, the porous membrane experiences physical compaction. In addition, polymeric membranes with low mechanical strength may fail at high operating pressure and during backwashing cleaning. To enhance the mechanical properties of a polymeric membrane, nanofillers are incorporated into polymers. It has been reported that the reinforcement of polymeric membranes with small quantities of nanofillers can significantly increase the mechanical properties of membranes [4]. Therefore, investigations on the accurate characterization and improvement in mechanical properties of polymeric nanocomposite membranes are crucial for the evaluation of their performance, durability and further development potential. Moreover, the morphology of polymeric nanocomposite membranes is an important parameter that needs to be emphasized as well. Porosity, pore size and pore size distributions have been shown to affect the mechanical behavior of polymeric membranes [5]. Porosity by itself lowers the mechanical strength of a polymer [6]. Accordingly, the coupled effects of nanofiller content with porosity must be examined carefully to achieve an acceptable compromise for sufficiently good mechanical strength and performance of the porous polymer nanocomposite-based membranes. During operation, porous polymeric nanocomposites are vulnerable to both thermal and mechanical loads. Therefore, the durability of the membranes is highly dependent on the mechanical performance under such operating conditions. In fact, the performance and durability of these materials under a given load greatly depend on their microstructure, elastic properties and thermomechanical strength.

**Figure 1:**
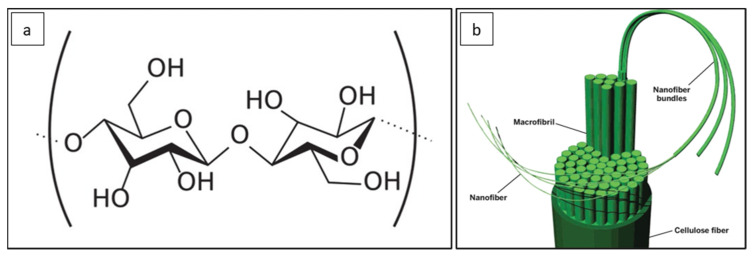
(**a**) Chemical structure of cellulose and (**b**) schematic of nanofibers derived from cellulose fiber (reprinted (adapted) with permission from [8]. Copyright (2015) American Chemical Society).

Cellulose is an abundant natural biodegradable polymer that exists in the form of micro and nano scale [7]. Figure 1 shows the chemical structure of cellulose [8]. Cellulose nanofibers (CNFs) are rich in hydroxyl groups, which makes them highly hydrophilic. CNF has been shown to improve the hydrophilicity, water flux and mechanical performance of PSF membranes [3,9,10,11]. It is important to mention that adding the appropriate amount of CNF is essential to achieve an improvement in the properties of the PSF membrane. Zhong and co-workers [12] showed that the addition of excess CNF to a PSF membrane caused the formation of very large voids at the bottom membrane surface that resulted in pore defects and irregular shape of finger-like pores in the cross-section. They determined that the formation of pore defects was a result of the agglomeration of nanofillers (i.e., CNF). Uneven dispersion in the casting solution is caused by the addition of excessive CNFs. This caused the formation of very large voids and pore defects in membranes [12,13]. Hence, the mechanical properties are lowered as the interfacial adhesion between the polymer and the nanofillers was weakened. Furthermore, since CNF is hydrophilic, the addition of excessive CNFs affects the kinetics of the phase inversion process during the membrane preparation. According to Zhong et al. [12], these conditions accelerated the phase inversion and also accelerated the growth of new-phase nucleus formation in the polymer-poor phase. However, when adding the appropriate amount of CNF, the mechanical properties in the PSF membrane were enhanced [12,13]. Consequently, controlling the composition of the casting solution is crucial as it substantially impacts the attained pore size, porosity and pore shape in a formed membrane. Zhang et al. [13] illustrated that the porous structure affected the water flux and the bovine serum albumin (BSA) rejection of membranes. Good connectivity in the finger-like pores resulted in increasing the pure water flux and, hence, improving the permeability of membranes.

Wang and co-workers [14] blended cellulose nanocrystalline (NCC) with PSF to improve the mechanical properties and hydrophilicity of a PSF hollow-fiber ultrafiltration (UF) membrane. They showed that the addition of a proper amount of NCC results in enhancing the tensile strength and elongation at the break. In addition, the pure water flux increased with increasing the NCC content from 0 to 1 wt.%; however, the bovine serum albumin (BSA) rejection decreases with the addition of NCC. Bai et al. [15] studied the effect of PSF, NCC and polyethylene glycol (PEG) content on the morphology, permeability, porosity and average pore size of the prepared composite membranes. It was shown that both porosity and average pore size increased with the increase in NCC content; hence, the permeability of the composite membranes also increased. It was concluded that the addition of 0.3 wt.% of NCC gave a large increase in the pure water flux compared to the pure PSF membrane (343.2 L/m^2^h (LMH) compared to 175.6 LMH) and maintained a high-BSA rejection ratio (more than 95%) [15]. Anokhina et al. [16] conducted a study where hollow-fiber UF PSF membranes were modified with CNF and it was shown that both the membrane permeability and the rejection of blue dextran increased.

In order to improve the mechanical properties of polymeric nano-filled membranes, it is a must to have well-dispersed nanofillers within the matrix [17]. However, there is a challenge in agglomeration associated with the introduction of nanocellulose into a polymeric matrix [17,18]. To reduce agglomeration, it is common that a hydrophilic additive, such as PEG and polyvinyl pyrrolidone (PVP), is added to the matrix polymer solution. These additives do not only increase the porosity and pore size of a membrane, they also increase the hydrophilicity and, hence, enhance the compatibility between the polymeric matrix and the CNF [18].

To the best of our knowledge, there is a need for research work that focuses on studying the properties of PSF membranes reinforced with CNFs, especially in terms of the mechanical properties. Most of the previous works on the preparation of a PSF membrane reinforced by CNF, via the phase inversion method, incorporated some sort of PSF–CNF interaction, since there is a challenge in incompatibility between the hydrophobic PSF and the hydrophilic CNF [9,12,13,19]. Zhong et al. [12] used sulfonated PSF (SPSF) to increase the compatibility between PSF and CNF. Zhang et al. [13] used surface modification for the CNF using a saline coupling agent to increase CNF compatibility with the PSF matrix. Both studies also used the additive PEG with a molecular weight of 400 and a content of 5 wt.% [12,13]. Hassan et al. [2] used rice straw for the preparation of CNF and then they incorporated the unbleached rice straw CNF into a PSF matrix using the phase inversion process. In this work, CNF, provided by the Process Development Center of the University of Maine, was successfully combined with the PSF matrix using the phase inversion method without surface modification of CNF or the use of an additive (e.g., SPSF, PEG, PVP, etc.).

In the present study, the aim is to fabricate PSF membranes reinforced with different loadings of CNFs and investigate their pore morphology, hydrophilicity and mechanical properties. The changes in the porous structure of the fabricated membrane with different CNF contents are evaluated. The stress–strain behavior for the synthesized PSF membranes with different CNF loadings is investigated. The effects of increasing the CNF content in the PSF matrix on the elastic modulus, yield stress and elongation at the break are studied. This is correlated to the dispersion of nanofibers within the membrane and their interaction with the PSF matrix.

## 2. Materials and Methods

### 2.1. Materials

In this study, polysulfone (PSF) membranes reinforced with cellulose nanofibers (CNFs) were fabricated. For the synthesis of membranes, PSF pellets (average molecular weight (Mw) ~35,000) and *N,N*-dimethylacetamide (DMAc, purity ≥ 99%) were purchased from Sigma-Aldrich (St. Louis, MO, USA). CNF was purchased from the Process Development Center of the University of Maine (Orono, ME, USA).

### 2.2. Membrane Preparation Method

First, 18 wt.% PSF was left to dissolve in 82 wt.% DMAc solution for 24 h at room temperature using a magnetic stirrer. CNFs with different loadings (0.1 wt.%, 0.2 wt.%, 0.3 wt.%, 0.5 wt.% out of PSF) were wetted using acetone. Vacuum filtration was then used to remove the acetone. DMAc was passed twice through the filtered CNF. The DMAc-wetted CNFs were added to the PSF/DMAc solution. A Q500 sonicator probe (Thomas Scientific, Swedesboro, NJ, USA) was used to ensure the homogenous dispersibility of CNFs in the PSF/DMAc solution. At the same time, continuous stirring was maintained using a labForce digital hotplate stirrer (Thomas Scientific, Swedesboro, NJ, USA) to further facilitate the dispersion of CNFs in the PSF/DMAc solution.

Before membrane casting, the casting solution was degassed in a water bath at 25 °C for 30 min to remove air bubbles. The membranes were prepared through the phase inversion method. The casting solution was poured onto a clean and dry glass plate and cast at room temperature using a casting knife (gap height of 200 µm) with a constant casting speed of 40 mm/s by employing an automatic thin-film applicator (TQC Sheen, Capelle aan den Ijssel, The Netherlands) After that, the glass plate was immersed into a coagulation bath filled with deionized (DI) water at room temperature. The membrane film was left to detach from the glass plate. Finally, the membrane was rinsed and stored in a plastic container containing DI water. Table 1 shows the composition of the fabricated membranes.

### 2.3. Characterization of CNFs and PSF/CNF Membranes

#### 2.3.1. Characterization of CNFs

The microstructure of CNFs is investigated through the use of scanning electron microscopy (SEM) with FEI Quanta 650 FEG (FEI, Hillsboro, OR, USA). Transmission electron microscopy (TEM) was also used to study the morphology of CNFs with FEI TalosF200X TEM (FEI, Hillsboro, OR, USA) at 200 kV.

#### 2.3.2. Characterization of PSF/CNF Membranes

Membrane Morphology

A field emission scanning electron microscopy (FE-SEM) technique was used to study the top surface and cross-section morphologies of PSFs and PSF/CNF membranes using FEI Quanta 650 FEG (FEI, Hillsboro, OR, USA) with a set vacuum condition at 3 kV. Liquid nitrogen was utilized to prepare the cross-section of the membrane samples before their coating with a 5 nm gold layer for electrical conductivity.

Water Flux

Pure water flux of the membranes was measured using a dead-end HP4750 stirred cell (Sterlitech, Kent, WA, USA). All membrane samples were cut into circular shapes using a circular mold with a diameter of 46 mm. Then, the samples were washed thoroughly with DI water to remove any impurities before the filtration test. To pressurize the feed solution in the filtration unit, nitrogen gas was utilized. The permeate flux, J, (LMH) was calculated using the following equation [20]:

(1) J=Q/(A×T)

here, Q is the permeate volume (L), A is the effective membrane surface area (m^2^) and T is the filtration time (h). All filtration tests were conducted at room temperature (25 °C).

Regarding the compaction of a membrane, the compaction factor (CF) for a membrane at a given operating pressure can be calculated using the below equation [21]:

(2) $$CF=\frac{J_{in}}{J_{st}}$$

here, $J_{in}$ and $J_{st}$ are the initial DI water flux and the DI water flux after 60 min of the filtration test, respectively (LMH).

Porosity, and Pore Size of Membranes

Porosities of all fabricated membranes were determined using the gravimetric method [10]. From each membrane, three samples were used. First, membranes stored in DI water were cut into circular shapes using a circular mold with a diameter of 46 mm. After gentle wiping of the three samples, the wet weight ($w_{w}$) for each sample was measured. The samples were then dried in an oven at 50 °C for 24 h. Samples were left for 10 min to cool down before taking their dried weight ($w_{d}$). The following equation was used to calculate the total porosity (ε) of each sample and then the average porosity for each membrane was determined [22]:

(3) $$\varepsilon =\frac{w_{w}-w_{d}}{A\times l\times \rho }\times 100\%$$

where A is the membrane surface area (m^2^), l is the thickness of the membrane sample (m), ρ is the density of DI water at 25 °C (g/m^3^) and $w_{w},\;w_{d}$ are the wet and dry masses (g) of the membrane sample, respectively.

To determine the average pore size of membranes, the Guerout–Elford–Ferry Equation was used [23]:

(4) $$r_{m}=\sqrt{\frac{8\eta l\dot{Q}\left(2.9-1.75\varepsilon \right)}{\varepsilon A\Delta P}}$$

here, η is the water viscosity at 25 °C (0.89 mPa.s), $\dot{Q}$ is the permeate volume per unit time (m^3^/s), P is the operating pressure (Pa), A is the membrane surface area (m^2^), l is the thickness of the membrane sample (m) and ε is the total porosity.

Water Contact Angles of Membranes

The water contact angle of fabricated membranes was measured utilizing a Kruss DCA-25 contact angle goniometer (Kruss GmbH, Hamburg, Germany). For each membrane, the contact angle was measured at two different locations with a 1.5 µL droplet size of DI water at 25 °C.

Mechanical Properties of Membranes

The mechanical properties of the fabricated membranes (both elastic modulus and stress–strain behavior) were characterized through Dynamic Mechanical Analysis (DMA) tests using DMA Q-800 supplied by TA Instrument (TA Instruments, New Castle, UK). The membrane sample is stretched uniaxially at a constant displacement rate of 200 µm/min while both ends are gripped until the sample breaks. The elastic modulus and yield stress are determined using the obtained stress–strain curves through TA universal analysis software. The elastic modulus is determined from the slope of the linear elastic region in the stress–strain curves, whereas the yield stress is determined from the onset point from the plastic deformation (the stress that corresponds to 3% strain). Three samples from each membrane were tested and analyzed to ensure the reliability of the results.

## 3. Results and Discussion

### 3.1. CNF Characterization

**Figure 2:**
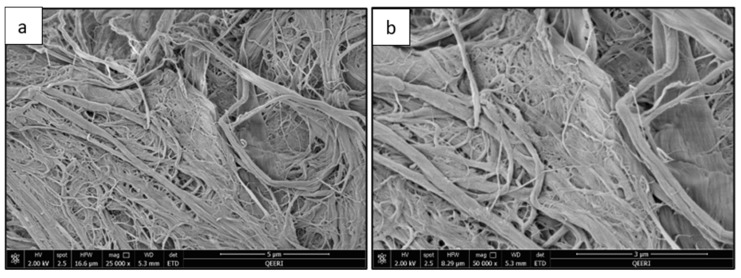
SEM images of CNF at (**a**) 5 µm and (**b**) 3 µm scale.

**Figure 3:**
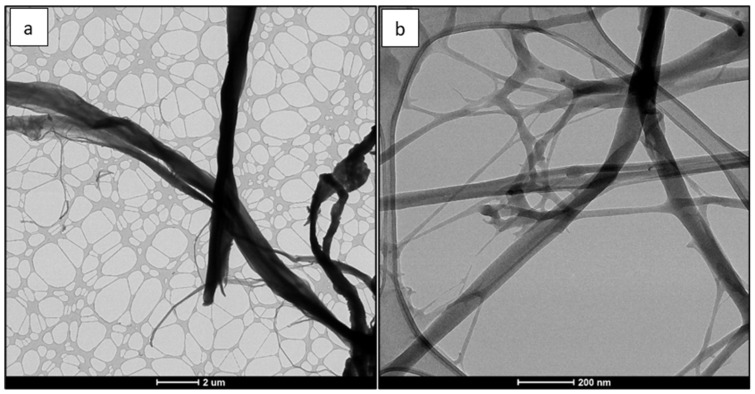
TEM images of CNF at two magnifications whose scale are: (**a**) 2 µm and (**b**) 200 nm.

Figure 2 illustrates the SEM images of CNFs and Figure 3 shows the TEM images of CNFs. The length and width measurements for CNFs are provided by the University of Maine (Process Development Center, Orono, ME, USA). The length of CNF ranges from 130 nm to 225 µm and the width ranges from 5 to 200 nm [24]. The range of aspect ratio is from 14 to 23. CNF has a relatively high aspect ratio; hence, CNF can be an excellent reinforcement to improve the mechanical properties of a PSF membrane.

### 3.2. Characterization of Fabricated Membranes

#### 3.2.1. Morphology of Membranes

**Figure 4:**
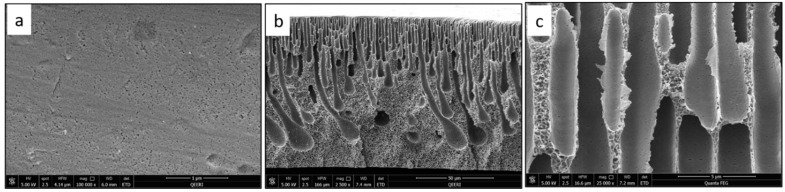
SEM images for pure PSF (M1) sample: (**a**) top surface, (**b**) cross-section, (**c**) enlarged top cross-section.

**Figure 5:**
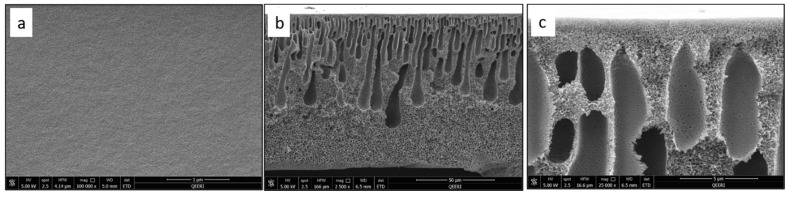
SEM images for PSF/0.1 wt.% CNF (M2) sample: (**a**) top surface, (**b**) cross-section, (**c**) enlarged top cross-section.

**Figure 6:**
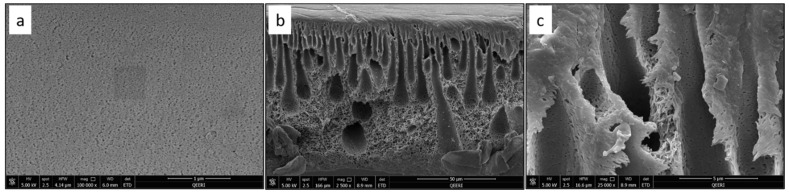
SEM images for PSF/0.2 wt.% CNF (M3) sample: (**a**) top surface, (**b**) cross-section, (**c**) enlarged top cross-section.

**Figure 7:**
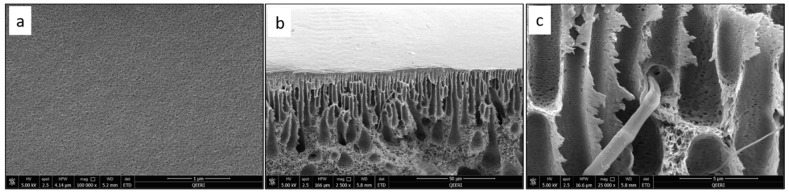
SEM images for PSF/0.3 wt.% CNF (M4) sample: (**a**) top surface, (**b**) cross-section, (**c**) enlarged top cross-section.

**Figure 8:**
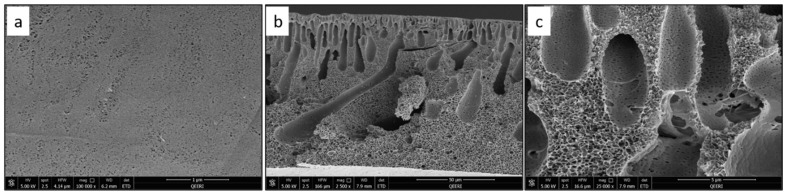
SEM images for PSF/0.5 wt.% CNF (M5) sample: (**a**) top surface, (**b**) cross-section, (**c**) enlarged top cross-section.

SEM characterization was performed for the fabricated PSF membranes with different CNF loadings to study their pore morphology. Figure 4 depicts the top surface and cross-section images of the pure PSF membrane. Figure 5, Figure 6, Figure 7 and Figure 8 demonstrate both the top and cross-section SEM images for PSF membranes incorporated through four different CNF loadings: 0.1 wt.%, 0.2 wt.%, 0.3 wt.% and 0.5 wt.%, respectively. The top surface images of membranes show a slight increase in pore size. As the CNF content increases in the PSF solution, the hydrophilicity of the casting solution increases. An increase in hydrophilicity of the casting solution results in faster de-mixing between the solvent (DMAc) and the nonsolvent (water) during the formation of a membrane [25].

**Figure 9:**
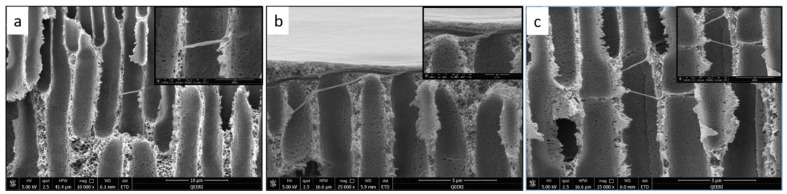
Illustration of the CNF in different SEM cross-section images for: (**a**) PSF/0.2 wt.% CNF (M3), (**b**,**c**) PSF/0.3 wt.% CNF (M4).

From the SEM cross-section images, it can be observed that membranes contain finger-like macro pores, which become longer with bigger macrovoids in the membrane’s bottom structure as the CNF content increases. In addition, the connectivity between the finger-like pores increases from M1 to M5 (0 to 0.5 wt.% CNF loading). The enlarged cross-section in Figure 7c clearly shows the reinforced cellulose nanofibers. More cross-section images illustrating the incorporated nanofibers within the different membrane samples can be seen in Figure 9.

#### 3.2.2. Porosity and Pore Size of Membranes

**Figure 10:**
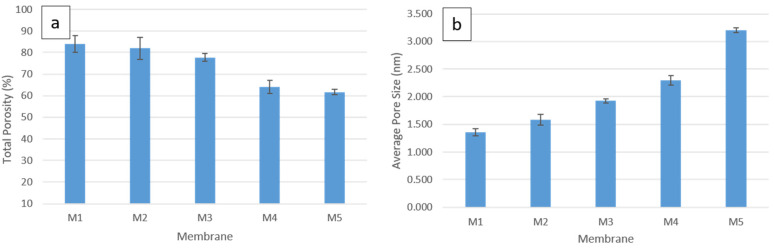
Pore morphology analysis for all fabricated membranes: (**a**) total porosity and (**b**) average pore size.

The gravimetric method, explained in the previous section, was used to determine the porosity of all fabricated membranes, which are displayed in Figure 10a. The average pore size for each membrane was also calculated and the results are shown in Figure 10b. It can be seen that the total porosity in the pure PSF membrane (84%) slightly decreases with the addition of 0.1 wt.% CNF (82%) and 0.2 wt.% CNF (78%) into the PSF matrix. Incorporating 0.3 wt.% CNF into PSF solution results in a greater decrease in the total porosity. The strong interaction between the PSF matrix and the nanofibers at low-CNF content might result in lowering the total porosity. The total porosity continues to decrease with the addition of 0.5 wt.% CNF, but with a minor decrease. On the other hand, the average pore size increases with the CNF reinforcement into the PSF matrix. The largest average pore size is found to be 3.2 nm for M5 (PSF/0.5 wt.% CNF), whereas the smallest average pore size is found to be 1.4 nm for M1 (pure PSF).

#### 3.2.3. Water Contact Angle

**Figure 11:**
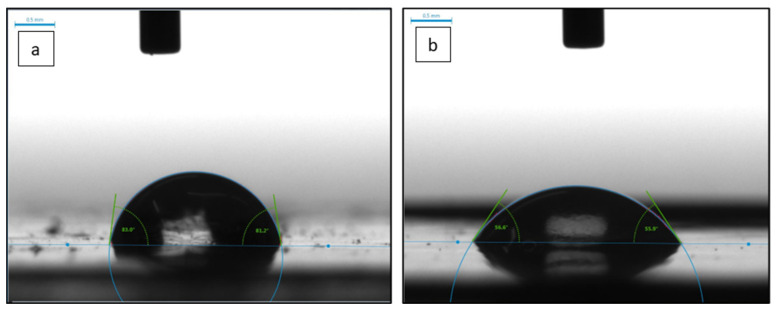
DI water drops images for: (**a**) PSF membrane (M1) and (**b**) PSF membrane with 0.3 wt.% CNF (M4).

Water contact angle (WCA) is an indication of the wettability and hydrophilicity of a membrane surface. As shown in Table 2, the pure PSF membrane gave the highest WCA among all other membranes, with a value of 86.7° ± 6.4°, which is expected due to the hydrophobic nature of PSFs. The WCA decreases to 63.5° ± 3.0° with the incorporation of 0.2 wt.% CNF and 57.9° ± 2.30° with the incorporation of 0.3 wt.% CNF. The large decrease in the WCA is explained by the hydrophilic nature of CNFs, as cellulose macromolecules contain many hydroxyl groups (Figure 1). However, the PSF membrane with 0.5 wt.% CNF loading (M5) has higher WCA than other PSF membranes incorporated with CNF. This could be due to the agglomeration of CNFs at higher CNF loadings. Agglomeration of CNFs results in decreasing the hydrophilicity of the membrane, as some areas contain clusters of agglomerated CNFs while other areas lack the presence of CNFs. The low WCA value is also an indication that the nanofibers are well dispersed and distributed within the PSF membrane incorporated with 0.3 wt.% CNF. Figure 11a,b demonstrate images of a water droplet at the surface of M1 and M4 membranes, respectively.

#### 3.2.4. Mechanical Properties of Membranes

**Figure 12:**
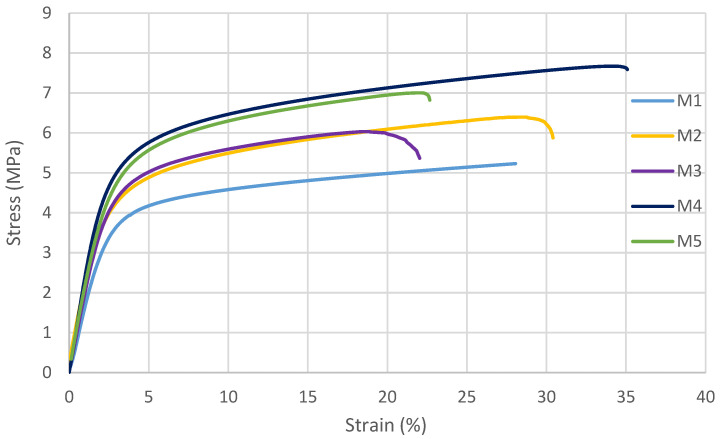
Stress-strain curves for the cast membranes.

Membranes are subjected to cycles of pressure and temperature changes during operation and cleaning; thus, it is necessary to investigate how the polymeric membranes behave under stress conditions [20]. In this study, CNFs were reinforced to enhance the mechanical properties of the PSF membrane. Figure 12 illustrates the stress–strain curves for the fabricated membranes. In general, it can be seen that the addition of CNF significantly improved the mechanical behavior of synthesized PSF membranes. The addition of 0.3 wt.% CNF to PSF matrix in M4 resulted in achieving the highest stress-strain curve. PSF membrane reinforced by 0.5 wt.% (M5) gave a significantly high stress-strain curve compared to M1, M2 and M3. At the same time, the stress–strain curve for M5 is slightly lower than the one for M4. This can be explained by the larger pore size under relatively similar porosities at a high-CNF loading (0.5 wt.%). This could also be due to uneven dispersion of the nanofibers in the polymer matrix, as CNFs tend to agglomerate, especially at high loading.

**Figure 13:**
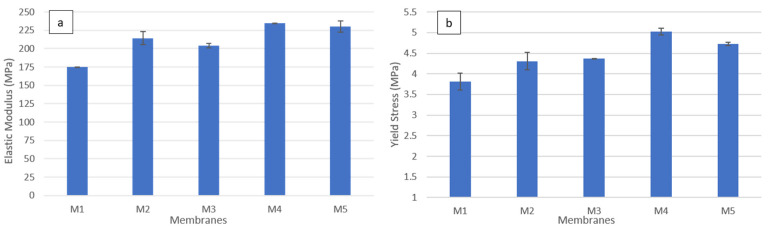
The mechanical properties of cast PSF membranes: (**a**) elastic modulus, (**b**) yield stress.

In terms of mechanical properties, the highest elastic modulus (the slope of the linear elastic region in the stress-strain curve) and yield stress (onset point of yielding) are achieved for the PSF membrane incorporated with 0.3 wt.% CNF, as shown in Figure 13. The elastic modulus increased by 34%, from 175.05 MPa for the pure PSF membrane to 234.5 MPa for the PSF membrane reinforced with 0.3 wt.% CNF. Similarly, the yield stress increased by 32%, from 3.81 MPa for the pure PSF membrane to 5.03 MPa for the PSF membrane reinforced with 0.3 wt.% CNF. Both the elastic modulus and yield stress slightly decrease with the addition of 0.5 wt.% CNF into the PSF matrix. As mentioned earlier, this can be explained by the higher membrane porosity and a higher tendency for agglomeration at a high-nanofiber loading.

**Figure 14:**
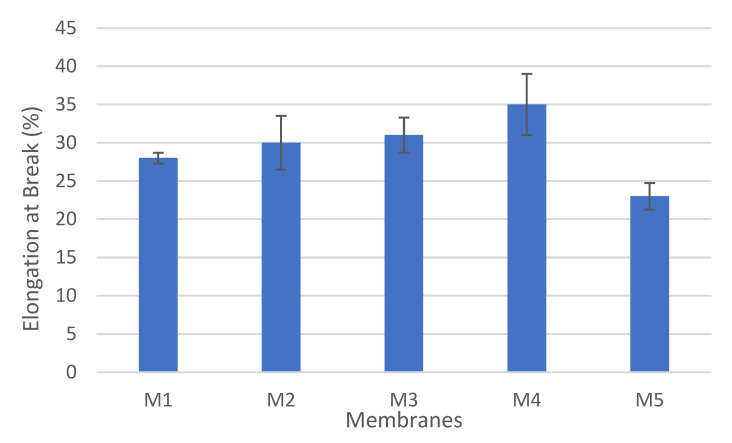
Elongation at break for cast PSF membranes.

Figure 14 depicts the changes in the elongation at the break ($(\Delta l/l_{i})\%\;$) with the addition of CNFs into the PSF matrix. The elongation at the break increased as the content of CNFs increased and reached its maximum value (35%) at CNF content of 0.3 wt.% (M4). Then, at CNF loading of 0.5 wt.% (M5), the elongation at the break decreased dramatically to 23%, which is even lower than that for the pure PSF membrane (M1(28%)).

The high aspect ratio of CNFs improves the interaction between the CNFs and the surrounding polymer matrices. A strong interaction between the nanofillers and the PSF matrix results in improved mechanical properties, including elastic modulus and yield stress [17].

#### 3.2.5. Water Flux

**Figure 15:**
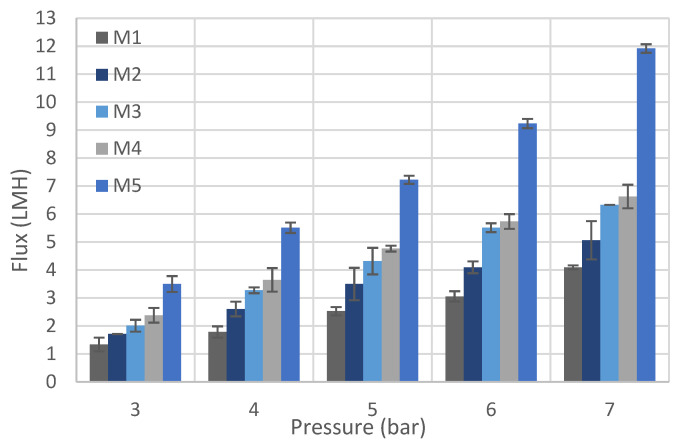
Pure water flux (PWF) for cast PSF/CNF membranes (test condition: room temperature, DI water).

Filtration tests, as explained in Section 2, were conducted on all fabricated membranes to investigate the permeate flux. Figure 15 depicts the changes in pure water flux (PWF) as the CNF loading in the PSF matrix increases from 0 (M1) to 0.5 wt.% (M5) at different operating pressures. A noticeable increase in the PWF values is observed as the CNF content increases from 0 (M1) to 0.3 wt.% (M4). For M5 (0.5 wt.% CNF), the flux increase is larger compared with the other membranes, especially at higher operating pressure values. These results are expected as the average pore size increases with higher CNF loading, as shown in Figure 10.

**Figure 16:**
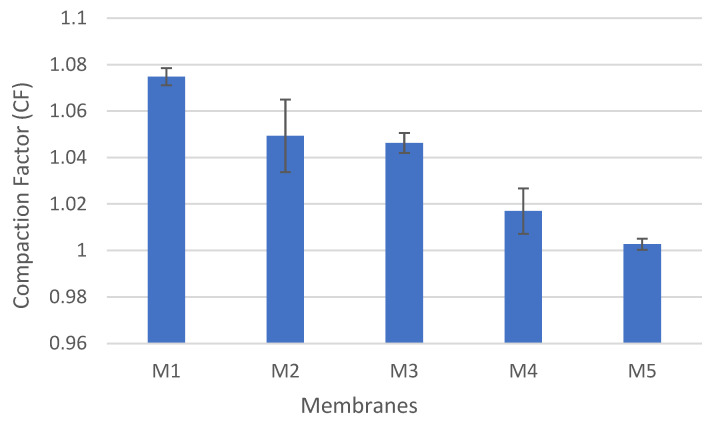
The CF values for PSF/CNF membranes at an operating pressure of 10 bar.

Moreover, the compaction factor (CF) for each membrane was determined, as presented in Figure 16. CF is an indication of the deformation severity in the membrane’s porous structure under applied pressure. The compaction factor decreases with the addition of CNFs into the PSF matrix. The CF is found to be 1.075 for pure PSF (M1), whereas M2, M3, M4 and M5 have CF values of 1.049, 1.046, 1.017 and 1.003, respectively. The incorporation of CNFs into PSF increases the resistance to compaction (i.e., lower CF values). This can be explained by the strong interaction between the PSF matrix and well-distributed nanofibers, which, as a result, improves the compaction resistance.

## 4. Conclusions

In this study, PSF membranes reinforced by different CNF loadings (0.1 wt.%, 0.2 wt.%, 0.3 wt.% and 0.5 wt.%) were synthesized through the phase inversion method. The effects of different CNF loadings on the pore morphology of the PSF membrane were observed through SEM characterization. In addition, the total porosity values and average pore size were determined through the gravimetric method and the Guerout-Elford-Ferry equation, respectively. It was shown that the addition of CNF into the PSF matrix decreased the total porosity and increased the average pore size of the prepared membranes. The total porosity decreased from 84% (M1) to 64% (M4) with the addition of 0.3 wt.% CNF into the PSF matrix. It was shown that CNF incorporation within the PSF polymer matrix enhances the hydrophilicity in PSF/CNF membranes. For example, the WCA value for PSF/0.3 wt.% CNF (M4) membrane was found to be 57.9° ± 2.3°, whereas the pure PSF membrane has a WCA value of 86.7° ± 6.4°. DMA tests showed a significant improvement in the mechanical performance of the PSF membranes reinforced with different CNF loadings. The highest elastic modulus and yield stress were achieved for the PSF/0.3 wt.% CNF (M4), with values of 234.5 MPa and 5.03 MPa, respectively.

## Figures and Tables

**Table 1:** Composition of fabricated PSF membranes.

Membranes	CNF Content (wt.%)
M1	0 (Pure PSF)
M2	0.1
M3	0.2
M4	0.3
M5	0.5

**Table 2:** Water contact angle (WCA) of the membranes.

Membrane	M1	M2	M3	M4	M5
WCA (°)	86.7 ± 6.36	76.3 ± 1.06	63.5 ± 2.97	57.9 ± 2.30	84.9 ± 3.22

## Data Availability

Not applicable.

## References

[1] Purkait M.K., Sinha M.K., Mondal P., Singh R. (2018). Introduction to membranes. Stimuli Responsive Polymeric Membranes.

[2] Hassan M., Zeid R.E.A., Abou-Elseoud W.S., Hassan E., Berglund L., Oksman K. (2019). Effect of Unbleached Rice Straw Cellulose Nanofibers on the Properties of Polysulfone Membranes. Polymers.

[3] Li S., Gao Y., Bai H., Zhang L., Qu P., Bai L. (2011). Preparation and characteristics of polysulfone dialysis composite membranes modified with nanocrystalline cellulose. BioResources.

[4] Wang K., Ahzi S., Boumbimba R.M., Bahlouli N., Addiego F., Rémond Y. (2013). Micromechanical modeling of the elastic behavior of polypropylene based organoclay nanocomposites under a wide range of temperatures and strain rates/frequencies. Mech. Mater..

[5] Alasfar R., Ahzi S., Wang K., Barth N. (2020). Modeling the mechanical response of polymers and nano-filled polymers: Effects of porosity and fillers content. J. Appl. Polym. Sci..

[6] Alasfar R.H., Ahzi S., Barth N., Kochkodan V., Khraisheh M., Koç M. (2022). A Review on the Modeling of the Elastic Modulus and Yield Stress of Polymers and Polymer Nanocomposites: Effect of Temperature, Loading Rate and Porosity. Polymers.

[7] Bai H., Zhou Y., Zhang L. (2014). Morphology and Mechanical Properties of a New Nanocrystalline Cellulose/Polysulfone Composite Membrane. Adv. Polym. Technol..

[8] Delgado-Aguilar M., Quim T., Pèlach M.À., Mutjé P., Fullana-i-Palmer P. (2015). Are cellulose nanofibers a solution for a more circular economy of paper products?. Environ. Sci. Technol..

[9] Ding Z., Liu X., Liu Y., Zhang L. (2016). Enhancing the Compatibility, Hydrophilicity and Mechanical Properties of Polysulfone Ultrafiltration Membranes with Lignocellulose Nanofibrils. Polymers.

[10] Qu P., Tang H., Gao Y., Zhang L.P., Wang S. (2010). Polyethersulfone composite membrane blended With cellulose fibrils. BioResources.

[11] Benhamou K., Kaddami H., Magnin A., Dufresne A., Ahmad A. (2015). Bio-based polyurethane reinforced with cellulose nanofibers: A comprehensive investigation on the effect of interface. Carbohydr. Polym..

[12] Zhong L., Ding Z., Li B., Zhang L. (2015). Preparation and Characterization of Polysulfone/Sulfonated Polysulfone/Cellulose Nanofibers Ternary Blend Membranes. BioResources.

[13] Zhang W., Zhong L., Wang T., Jiang Z., Gao X., Zhang L. (2018). Surface modification of cellulose nanofibers and their effects on the morphology and properties of polysulfone membranes. IOP Conf. Series: Mater. Sci. Eng..

[14] Wang X., Bai H.L., Zhang L.P. (2012). The Effects of Nanocrystaline Cellulose on Polysulfone Hollow-Fiber Ultrafiltration Membrane. Adv. Mater. Res..

[15] Bai H., Wang X., Sun H., Zhang L. (2014). Permeability and morphology study of polysulfone composite membrane blended with nanocrystalline cellulose. Desalination Water Treat..

[16] Anokhina T.S., Bazhenov S., Borisov I.L., Vasilevsky V., Vinokurov V., Volkov A. (2019). Nanocellulose as Modifier for Hollow Fiber Ultrafiltration PSF Membranes. Key Eng. Mater..

[17] Jaffar S.S., Saallah S., Misson M., Siddiquee S., Roslan J., Saalah S., Lenggoro W. (2022). Recent Development and Environmental Applications of Nanocellulose-Based Membranes. Membranes.

[18] Malakhov A.O., Anokhina T.S., Petrova D.A., Vinokurov V.A., Volkov A.V. (2018). Nanocellulose as a Component of Ultrafiltration Membranes. Pet. Chem..

[19] Chakrabarty A., Teramoto Y. (2018). Recent Advances in Nanocellulose Composites with Polymers: A Guide for Choosing Partners and How to Incorporate Them. Polymers.

[20] Antolín-Cerón V.-H., González-López F.-J., Astudillo-Sánchez P.D., Barrera-Rivera K.-A., Martínez-Richa A. (2022). High-Performance Polyurethane Nanocomposite Membranes Containing Cellulose Nanocrystals for Protein Separation. Polymers.

[21] Kamal N., Ahzi S., Kochkodan V. (2020). Polysulfone/halloysite composite membranes with low fouling properties and enhanced compaction resistance. Appl. Clay Sci..

[22] Yuliwati E., Porawati H., Elfidiah E., Melani A. (2019). Performance of Composite Membrane for Palm Oil Wastewater Treatment. J. Appl. Membr. Sci. Technol..

[23] Arumugham T., Amimodu R.G., Kaleekkal N.J., Rana D. (2019). Nano CuO/g-C3N4 sheets-based ultrafiltration membrane with enhanced interfacial affinity, antifouling and protein separation performances for water treatment application. J. Environ. Sci..

[24] (2022). Cellulose Nanofibril Safety Data Sheet.

[25] Kamal N., Kochkodan V., Zekri A., Ahzi S. (2019). Polysulfone Membranes Embedded with Halloysites Nanotubes: Preparation and Properties. Membranes.

